# Improvements in Function With Inpatient Rehabilitation in a Patient Following a Traumatic Brain Injury: A Case Report

**DOI:** 10.7759/cureus.67538

**Published:** 2024-08-22

**Authors:** Stephen Howard, Ryan Tam, Nithyanandini Namassivaya

**Affiliations:** 1 Physical Medicine and Rehabilitation, Lake Erie College of Osteopathic Medicine, Elmira, USA; 2 Internal Medicine, Lake Erie College of Osteopathic Medicine, Elmira, USA; 3 Physical Medicine and Rehabilitation, Rochester Regional Health, Rochester, USA

**Keywords:** physical medicine and rehabilitation, interdisciplinary collaboration, optimizing function, acute inpatient brain injury rehabilitation, traumatic brain injury

## Abstract

Traumatic brain injury has been a leading cause of morbidity, mortality, and disability. Patients may experience cognitive and functional decline, depending on the severity. In this case report, a patient presents with a closed-head traumatic brain injury that was sustained after a motor vehicle accident. Through a comprehensive inpatient rehabilitation plan with physical therapy, speech therapy, occupational therapy, and neuropsychological assessments, this patient was able to gain functionality, as shown by his Ranchos Los Amigos Revised Scale. This scale is used to describe the cognitive and behavioral patterns found in recovering patients following a traumatic brain injury. With this case report, we hope to raise awareness within the medical community of the benefits of inpatient rehabilitation so that patients suffering from traumatic brain injury can receive better functional and cognitive recovery.

## Introduction

Traumatic brain injury (TBI) has been a leading cause of morbidity, mortality, and disability, with an incidence of 50 million individuals affected globally each year [1]. There are three primary categories of TBI: closed head, penetrating, and explosive blast. Closed-head TBI commonly occurs by blunt trauma mainly from motor vehicle accidents, falls, and sports activities. Penetrating TBI results from a foreign body that comes in contact with the brain parenchyma via a direct injury that passes through the skull region. Explosive blast TBI, which typically occurs in war-related events, results from rapid pressure shock waves that get transmitted from the skull into the brain parenchyma [1]. Although there may be noticeable effects right away, there are lasting effects that may occur and progress gradually.

On initial assessment of a patient with TBI, the Glasgow Coma Scale (GCS) is oftentimes used to assess the extent of impaired consciousness in patients by assessing responsiveness to eye-opening, motor, and verbal responses, graded on a scale with total values between 3 (worst) to 15 (best). GCS is important as it can provide clinical decisions to not only triage the severity of the patient but also help guide treatment management such as whether the patient should have their airway secured [2]. In addition to the GCS in assessing acute medical and trauma patients, the Ranchos Los Amigos Revised Scale (RLAS-R) is used in conjunction with the initial evaluation and during the subsequent recovery period. RLAS-R takes into account the patient’s level of consciousness and their reliance on assistance to carry out cognitive and physical functions, graded in the different levels from one (no response - total assistance) to ten (purposeful, appropriate - modified independently) [3]. The RLAS-R scale is also helpful when assessing the patient's recovery status as it emerges from a comatose state which can help plan for rehabilitation [3].

Three principles have historically guided neurorehabilitation: reversal of diaschisis, behavioral compensation, and neural plasticity [4]. Inpatient rehabilitation facilities play an important role in caring for patients who have undergone TBI, which differs from other long-term acute care or skilled nursing facilities. In inpatient rehabilitation facilities, physiatrists often coordinate the care amongst the interdisciplinary team members with a minimum of 15 hours per week of intensive therapy for acutely ill individuals [4]. Therefore, patients are two times as likely to be discharged back into the community and oppose living in a skilled nursing facility [4]. In addition, inpatient rehabilitation has a rehospitalization rate that is one-third less than those receiving care in skilled nursing facilities [4]. 

In the following case report, we highlight a patient who suffered a TBI that received inpatient rehabilitation and successfully recovered functionality so that he can perform basic needs.

## Case presentation

A 35-year-old male presents to the inpatient rehabilitation unit following a traumatic brain injury. His past medical history was significant for right shoulder cuff injury, chronic lower back pain in the L4-S1 dermatome, multiple concussions, and right knee injury playing lacrosse. He was previously admitted to a nearby hospital following a severe TBI from being hit by a car. The patient worked for law enforcement and was arresting someone in the parking lot of a store when the person attempted to flee, hitting the patient with his vehicle going approximately 20 miles per hour. The patient was hit at the hood of the car, flipped over the vehicle, and fell to the ground, striking his head and immediately began having a seizure. The patient was found by emergency health services as unresponsive with palpable pulses, bradycardic in the 40s, and seizure-like activity. 

**Figure 1:**
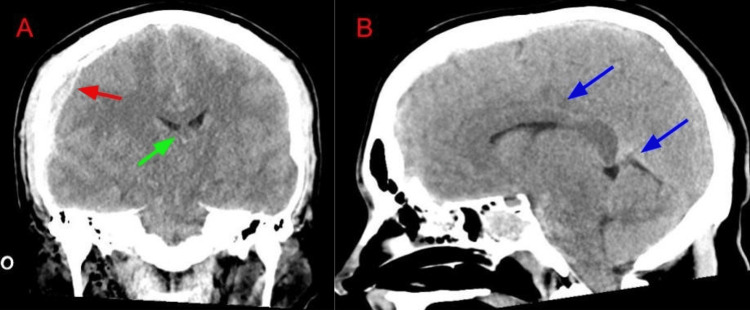
Noncontrast CT head coronal (A) and sagittal (B). Comminuted fracture of the right temporal bone, extending to the right occipital bone. Large subdural hematoma along the right cerebral convexity (red arrow), causing midline shift to the left by approximately 8 mm (green arrow). Diffuse subarachnoid hemorrhage (blue arrows).

He arrived at the emergency department approximately 20 minutes following the incident with a GCS of 3 with minimal respirations. The patient was then intubated with an endotracheal tube for airway control. Vital signs were remarkable for bradycardia, hypertension, and bradypnea, consistent with Cushing’s Triad following significant intracranial injury. A Focused Assessment with Sonography in Trauma (FAST) exam was done which was negative. On exam, the patient had a scalp laceration along the right side of the head with bogginess. His right pupil was 7 mm, and his left pupil was 6 mm, both eyes nonreactive to light. The patient had a c-collar in place, multiple skin lacerations present on his bilateral elbows, and road rash abrasions to the lumbar and sacral back. The patient was unresponsive with decerebrate posturing. The patient was given intravenous fluids and brought for computerized tomography (CT) imaging of the head. The CT showed a right temporal bone fracture, right-sided subdural hematoma with midline shift, and scattered subarachnoid hemorrhage (Figure 1). He was taken by the flight crew directly from the CT scanner and transported to a level one trauma center for higher level care.

On arrival at the level one trauma center, the patient was seen by neurosurgery for intraparenchymal pressure monitor placement and right frontal twist drill craniotomy for evacuation of the hematoma. The neurosurgeon followed this patient for intracranial pressure measures including mannitol, hypertonic saline, and anticonvulsant prophylaxis. The patient also showed signs of early paroxysmal sympathetic activity and was given Tylenol and cooling blankets. The following day, the patient neurologically became more responsive. The following week, the patient remained stable, extubated, and started some bedside physical therapy. The patient was then discharged two weeks later to acute inpatient rehabilitation for comprehensive rehabilitation.

When the patient arrived at the inpatient rehabilitation unit, he was somnolent, yet arousable. On physical exam, the patient had sutures in the anterior head, oral thrush, and a foley catheter present. Neurologically, the patient had right eye ptosis, disconjugate gaze, and right-sided facial droop. He opened his eyes spontaneously and answered a few simple questions with soft speech. His Modified Ashworth Scale (MAS), a scale from zero to five to measure spasticity, was zero and his RLAS-R was Level IV. He was diagnosed with severe TBI with impaired mobility, self-care, dysphagia, speech, and cognition. His plan was to begin acute comprehensive rehabilitation with physical therapy (PT), speech therapy (ST), occupational therapy (OT), and neuropsychology evaluation. The patient arrived agitated, impulsive, and lacking insight. Thus, he required a posey bed due to his increased risk for falls.

It has been shown that inpatient rehabilitation significantly improves functional independence measures in patients who experience TBI, most evident in motor recovery [5]. The patient’s functional recovery is noted in Table 1.

**Table 1:** Functional status of the patient on weekly basis. The functional progress of this patient can be analyzed using the Ranchos Los Amigos Revised Scale (RLAS-R). This scale is used to describe the cognitive and behavioral patterns found in patients following a traumatic brain injury as they recover from the injury. The score is graded from I (no response with total assistance) to X (purposeful, appropriate with modified independence). This patient's functional abilities improved from level IV to levels VI-VII in four weeks.

Length of stay (week)	Physical therapy	Occupational therapy	Speech therapy	Neuropsychology assessment	RLAS-R score
0	Global muscular weakness, difficult to produce on command due to apraxia. The patient needs three assists for transfers using Apex.	Significant deficits in attention and safety awareness. Decreased range of motion of the upper body, abnormal tone, agitation, impulsivity, decreased endurance, strength, balance, and coordination.	Language of confusion. Tolerating mechanical soft/thin diet with feeding assistance. Right-sided facial weakness resulting in mild right-sided buccal pocketing. Severe short-term memory impairment.	Substantial impairment of orientation, memory, and insight. Patient is currently in post-traumatic amnesia following traumatic brain injury.	Level IV
1	Ambulation with two assists and a rolling walker short distances. The patient continues to have decreased bilateral knee control and decreased motor planning for all standing, transfers, and stepping.	The patient is making functional progress as he demonstrates improved activities of daily living, independence, right upper extremity function, and visual processing. He is limited with executive skills/organization and working memory.	Significantly less agitation and restlessness. Continues with moderate-to-severe cognitive/linguistic impairments impacting his ability to fully express basic wants and needs. He makes gains in sustained attention and continues to require minimal cues for redirection at times.	He is better oriented to the place, but not the date. He is not oriented to his age and does not remember recent events. He is less tangential and somewhat confused.	Levels IV-V
2	Ambulation with one assist with the rolling walker and without a device. He continues with decreased right knee control and decreased motor planning for all standing, transfers, and stepping. He notes some increased dizziness and diplopia throughout mobility.	Patient has improvement in functional progress and visual processing. He is limited in executive skills and organizational skills due to diplopia.	Improvement in breath support with less hypophonia noted. He is improving in his ability to complete open phrase tasks with less confabulations. He continues to have reduced insight and short term memory. However, he is showing an emerging ability to recall new information. The patient is making gains in voluntary movement of right-sided facial musculature.	He was oriented to person, age, date of birth, city, home address, month, year, and place. He was not oriented to aspects of date/time or recent events requiring new learning.	Level VI
3	Patient has made excellent progress since his initial evaluation and has met all 5 out of 5 short-term goals for the week. He has improved balance and mobility. Patient was positive for benign paroxysmal positional vertigo with some improvement in diplopia and dizziness.	Patient has improvement in functional progress and visual processing. He has some limitations with executive skills/problem solving, and working memory.	Patient has improved his ability to answer personal questions and participate in basic conversation with intermittent confabulations. He is able to carry over conversations with mild reduction in recall. Patient is now out of post-traumatic amnesia and is oriented to person, place, time, and event.	Patient has improved considerably and is oriented to person, place, time, and event. He has good language and communication skills despite residual impairments of memory, executive function, and psychomotor speed.	Levels VI-VII
4	Patient has met 6 out of 7 short-term goals for the week. He has improved his standing balance, all mobility skills for bed mobility, transfers, and standing. He also improved ambulation and stairs endurance.	Patient has improvement in functional progress, core strength, visual scanning, and visual processing. Patient has some limitations with executive skills/problem solving, and working memory.	Patient is oriented to person, place, time, and event. He has significant gains in sustained and alternating attention at this time. Small gains in awareness of cognitive deficits noted, however, he continues with reduced insight into physical limitations with reduced safety awareness.	He is doing exceptionally well emotionally and behaviorally especially given his difficult circumstances and the losses and stresses this injury has caused for him and his family. He was communicating effectively and attending to a task well in a distracting environment. He is remembering more pragmatic information day by day.	Level VI-VII

As in the case described above, the patient improved their RLAS-R score from level IV to level VII within four weeks. With the help of physical therapy, occupational therapy, speech therapy, and neuropsychology, this patient was able to regain a significant amount of his function that he previously had before his accident. The patient initially presented with global weakness, significant deficits in attention, confusion, and was in post-traumatic amnesia. Within four weeks of inpatient rehabilitation, the patient made significant improvements in core strength, balance, mobility, orientation, and communication.

## Discussion

Initially, this patient presented with a high risk for falls due to his agitation which frequently occurred during the nighttime. His wife noted that she was often fearful of him falling at night and was struggling to stay awake enough to observe him throughout the night. Shared decision-making was made to establish him with a Posey bed to prevent him from having a fall during the night. Managing agitation in patients recovering from traumatic brain injury can be challenging and is one of the most clinically significant sequelae observed [6]. Approximately 50% of patients who experience TBI have agitated behaviors, such as restlessness and impulsivity, likely due to post-traumatic amnesia [6]. Formal assessment, tools, and guidelines are not well established in caring for patients who experience agitation during early TBI recovery [6]. Some studies suggest the use of pharmaceutical agents; however, non-pharmacological treatment is recommended as the first-line approach [6]. In an interventional survey, it has been found that 13% of providers use Posey beds in managing agitation secondary to TBI [6]. This patient was placed in a Posey bed at night for four weeks, which significantly reduced his fall risk. He also received neurostimulants from the on-staff physiatrist, including modafinil, to help improve his agitation, alertness, attention, focus, and concentration. With the significant improvement noted in neurocognitive function and the reduction in his agitation status, the Posey bed was eventually discontinued.

Patients who experience moderate to severe TBI require multiple different types of intervention to assist in their recovery, such as physical, cognitive, emotional, and behavioral. Inpatient rehabilitation for patients who experience TBI is effective and cost-efficient [7]. However, approximately 13% to 25% of patients who endure a TBI receive comprehensive inpatient rehabilitation, and even fewer receive TBI-specialized rehabilitation [7]. Rather, these patients are often discharged home, with or without home care, or to a skilled nursing facility (SNF) [7]. SNF may not provide the specialized therapy that is often required for these patients with TBI, as some of these facilities only offer physician visits as frequently as once a month [7]. The primary determinant of whether a patient with TBI receives inpatient rehabilitation or SNF is healthcare insurance coverage, as rehabilitation services generally require prior authorization by health insurance payers [7]. A major barrier to insurance companies approving patients for comprehensive inpatient rehabilitation is the *three-hour rule* [8]. This rule requires patients to have sufficient energy and endurance to be able to participate in occupational, speech, or physical therapy for three hours a day, five days a week [8]. SNF does not require this rule, thus acute care facilities may choose to discharge patients with TBI to SNF, even though the patient may benefit more from inpatient rehabilitation [7]. This rule has been shown to lack evidence that three hours of therapy per day is a significant driver in patient outcomes in inpatient rehabilitation [7]. 

This case presents an important discussion regarding the use of inpatient rehabilitation for patients with TBI. Patients, such as the one described above, have shown significant improvement in functional outcomes. Thus, insurance protocols for consideration of inpatient rehabilitation need to be revisited. 

## Conclusions

TBI has been a leading cause of morbidity, mortality, and disability. In this case report, we highlight a 35-year-old male who was presented to the inpatient rehabilitation unit after TBI and demonstrated significant improvement in clinical, consciousness, and functional status with the aid of rehabilitation. Inpatient rehabilitation plays a vital role in patient recovery as they experience a comprehensive program of physical therapy, speech therapy, occupational therapy, and neuropsychological assessment. With this case presentation, we hope to inform medical providers of the benefits of inpatient rehabilitation to allow safer discharge planning for patients for better functional and cognitive recovery. 
